# Revisiting Women Empowerment Through a Cultural Lens a In-Depth Analysis of Empowerment Methodologies in Horticulture in Rural Ethiopia

**DOI:** 10.3389/fpsyg.2021.536656

**Published:** 2021-11-24

**Authors:** Sarah De Smet, Smaranda Boroş

**Affiliations:** ^1^SNV Netherlands Development Organisation, The Hague, Netherlands; ^2^Vlerick Business School, Ghent, Belgium; ^3^Faculty of Economics and Business Administration, Department of Marketing, Innovation and Organisation, Ghent University, Ghent, Belgium

**Keywords:** women empowerment, power distance, participative methodologies, implicit bias, tight cultures, power relations, Global South

## Abstract

As previous research in international development has clearly demonstrated (see Banerjee and Prasad, 2008 for an overview), cultural values have an impact on the conceptualization of empowerment. In this paper we explore the implications of Power Distance as a cultural dimension for the use of participatory methodologies toward achieving women empowerment in rural areas in the Global South. Our critical analysis of cultural differences between the intervention facilitator (a Western-based NGO) and a rural community in SNNPR (Southern Nations, Nationalities and People’s Regions) in Ethiopia reveals how discrepancies in the perception of cultural values impacted the different stages of the intervention. These discrepancies ranged from the principles of facilitation (facilitation from the back and its paradoxical effects in such hierarchical contexts) to the focus on tools (on equality between individuals rather than focus on the family as the smallest unit). Discrepancies also surfaced from the selection criteria of participants (highly vulnerable groups; one spouse per family; number of participants from one community all of which prevented the impact of the intervention to be more powerful in the long run) and from how the participants are organized during trainings (the ratio of mixed vs. segregated groups and the criteria of group segregation – this can play a large role in regard to the potential openness of conversations and the creation of safe spaces to explore new identities which are the key to empowerment). Through all the stages of the intervention, we make suggestions on how to better implement such methodologies in the future, in a context-sensitive manner, by considering the cultural differences in assumptions and practices.

## Introduction

International development initiatives fall broadly under two umbrellas: traditional and decolonial approaches. The former, stemming from neoliberal Western thinking (Mohanty, 1995), are generally based on the assumption that access to and involvement in a capitalist economy engenders liberatory effects that lead to an increase in agency and well-being. Within this paradigm, empowerment is defined as the “process by which those who have been denied the possibility to make strategic life choices acquire such an ability” (Kabeer, 1999). Decolonial approaches on the other hand, strive to redefine the very notion of empowerment beyond an economic and individualist-based view, in a way that challenges global systems of oppression (Kurtiş et al., 2016). Decolonial approaches bring a much-needed radical rethinking of empowerment from both a philosophical and pragmatic standpoint, and have been making marked progress toward supporting “ways of being that produce broader empowerment and sustainable well-being within the cultural ecologies of embedded interdependence that constitute the typical habitat for the marginalized majority of humanity” (Kurtiş et al., 2016, p. 387). Nonetheless, traditional approaches still dominate public discourse (including UN goals^1^) and policies of international development institutions.

Within this mainstream (traditional) theorizing of empowerment, gender equality (defined as both men and women having equal rights, access to social goods, services, resources and opportunities – Kabeer, 2011) is seen to have a positive impact on economic and inclusive growth (Kabeer, 2016). While empowerment strategies strengthen the individual agency of women to challenge gender inequality, the assumption is that it is impossible for them to employ this agency as long as the structures that create and reproduce gender inequality remain the same (Kabeer, 1999). Subsequently, international development institutions operate under the belief that external influence is needed for empowerment to occur (Luttrell et al., 2009). These interventions, however, stem from a very specific (albeit hegemonic) cultural discourse, and perpetuate neoliberal and individualistic conceptualizations of empowerment (Adams et al., 2015). Such hegemonic cultural assumptions therefore need to be challenged (even from within the traditional empowerment paradigm), to reveal – and eventually address – the cultural biases behind development interventions.

In this paper, we discuss the implications of two cultural dimensions – tightness (Gelfand, 2019) and power distance (Hofstede, 1984) – on the design, implementation, and evaluation of an NGO-led gender empowerment intervention in rural Ethiopia that used participatory methodologies^2^. Tight cultures are characterized by clearly defined and reliably imposed social norms, leaving little room for individual improvisation and interpretation (Gelfand, 2019). In general, tight cultures have less gender egalitarianism and more traditional gender norms and roles (Toh and Leonardelli, 2012), with men having more access to power (social, economic, informational) and women bound to family roles (Drucza and Abebe, 2017). In such societies, women have less access to education, fewer possibilities to earn their own income, and more family duties. Consequently, they have more constraints in their life choices, or, according to the traditional paradigm, are less empowered (Kabeer, 1999). Women empowerment faces a doubly difficult challenge to emerge in such societies: on the one hand adherence to norms is very strict and breaking them comes at a high price (Curşeu et al., 2012; Stamkou et al., 2019); on the other, those with more power in the social structures have no interest in changing those norms. In other words, women empowerment is hindered by two dimensions which define culture: tightness/looseness of norms (Gelfand, 2019) and power distance (Hofstede, 1984).

These dimensions are particularly relevant for participatory methodologies, which are widely spread in mainstream development interventions. These interventions are based on assumptions of individualism and aiming to achieve equality as the desirable end. Therefore, it is through their very design that they challenge the existing power structures and cultural norms. Within a paradigm that conceptualizes development as “people becoming or being helped to become conscious about themselves and their environment, after which plans and actions are expected to follow, the involvement of people in the process of helping themselves is a cornerstone of good development and their awareness of this explains why development organizations have attached so much importance to participatory methodologies” (Ngunjiri, 1998 p. 466). Not surprisingly then, recent calls to gain deeper, more nuanced and contextualized understanding on the *effectiveness* of participatory methodologies have been made in most international development journals (e.g., European Journal of Development Research; Impact Assessment and Project Appraisal).

Therefore, the current paper sets out to paint, through ‘thick description’^3^ (Geertz, 1973), a nuanced image of how the cultural assumptions embedded in the design of a participatory empowerment intervention facilitated by a European NGO^4^ and those embedded in the local community converge and diverge to impact the effectiveness of the intervention. As the recipient (a rural community in Ethiopia) is a tight culture with high power distance structures and strong normative context, we focused primarily on the dimension of power distance in our analysis of the interplay between design and delivery principles of the intervention and the local community.

Our research contributes to the ongoing conversation on development interventions in three ways. First, remaining within the traditional theorizing of empowerment, it takes a nuanced approach in the cultural analysis of the intervention, seeing both parties (intervention facilitator and community/receiver) as partners in the empowerment intervention. Most research within this paradigm so far fails to zoom in on the cultural assumptions of the intervention facilitator and highlight the complex dynamics of the interaction, with implicit assumptions on both sides translating into action and reaction (Gray, 2008). To this end, we use the van Tulder et al. (2016) model of cross-sector partnerships to analyze, along the various stages of the collaboration, both the facilitator’s and the community’s actions and reactions, and explore the assumptions behind these. Second, we opted for a longitudinal qualitative methodological approach to our case study, in order to be more able to reveal these assumptions, bring to light complex dynamics among actors, and explore how these impacted the effectiveness of the intervention, not just short term (in the immediate outcomes), but also longer term (in impact – according to van Tulder’s taxonomy, presented below in Section “Methodology and research context”). Only by using a longer-term approach to data collection, and a combination of methods and techniques for collecting the data, can we hope to build deeper, more nuanced, and contextualized understanding into how to design and implement sustainable development interventions. Third, we step away from the assumption of the researcher as an objective observer, and instead interact with the participants in their context and in time. One of the authors of this paper was the project manager for the intervention; the other is an academic specializing in cultural diversity and system dynamics. One author was born and raised in an egalitarian, not very tight culture; the other was born and raised in a tight, high power distance culture. Instead of seeing the complexities our own stories and roles bring to the research as limitations that need mitigating, we used them to challenge our own assumptions and interpretations, and explore and test in action our hypotheses and questions (Boroş, 2020). We opted for a thick description method of both collecting and analyzing our data, and try, in the spirit of this method, to present our results as ‘thick description’ (Geertz, 1973), in order to share with the reader not just the content but also the context of our findings. In so doing, we aim to spur additional questions and the desire to explore further the intercultural dynamics of development interventions, with the final aim to co-create more sustainable development interventions.

## Theoretical Background

### Empowerment as a Process

In line with Kabeer (1999), we define empowerment as a “process by which those who have been denied the possibility to make strategic life choices acquire such an ability”. For this ability to be exercised, it inevitably means that the causes of disempowerment are addressed not only through increasing choice and agency at the individual level, but also by tackling structural inequalities in societies (Luttrell et al., 2009). If unequal power relations have been internalized however, choice can be a reflection of the social expectations of the wider community rather than a consequence of the emergence of a critical perspective on those expectations and structural inequalities are being reinforced (Kabeer, 1999). Therefore, existing power relations might make it unrealistic for the disempowered to tackle inequality and disempowerment on their own and change is assumed to occur with facilitation from outside (Luttrell et al., 2009). At the same time, external facilitators are not supposed to impose their own cultural values or assumptions about empowerment in the process itself (Kabeer, 2011). Therefore, they need to analyze their own conceptualization of empowerment, to avoid possible cultural and value-based tensions (Luttrell et al., 2009). To increase the impact of the intervention, facilitators would need to approach the social change as open-ended (Kabeer, 1999), hence allowing indicators of success to reflect the desired shift in power structures from the community and not necessarily prescribed by the facilitator’s own interpretation of gender equality. This is where the cross-cultural differences on the dimension of power distance (Hofstede, 1984; Eylon and Au, 1999) come into play. Funding bodies and aid/intervention providers are mainly based in the US and Western Europe, in more egalitarian (low power distance) societies, whereas beneficiaries are often in hierarchical (high power distance) societies. Power distance, being one of the deeply entrenched cultural dimensions that organize the very structure of a society and the values around power distribution, becomes a ubiquitous influence in such empowerment interventions. The differences between facilitators and beneficiaries leads to conflicting definitions of both what changes should be made and the criteria used to assess the success or failure of an intervention. In the next section, we look into more detail at the attitudinal and behavioral consequences that power distance presents in a society, specifically in relation to empowerment processes.

Before we deepen the concept of power distance, we align with African postcolonial feminism that views power as relational and dynamic, framed within a certain cultural, historical and generational context (Blay, 2008). The current conceptions of empowerment are mainly a product of WEIRD societies (Henrich et al., 2010), hence previous research has mainly studied women as individual agents without considering the social relationships they are part of and that influence their behavior and adherence to gender norms (Huis et al., 2020). In other words, prevailing gender norms in a specific cultural context influence how women experience empowerment (Huis et al., 2017). The Participatory Action Learning for Sustainability (PALS) intervention discussed in this paper was considered by the facilitators to hold the potential for increasing gender equality and women empowerment. However, as the assumptions underlying this participative methodology are based upon Western norms and conceptualizations of empowerment, we need to be extremely cautious to interpret the results of the intervention and shed light on the insights from the experience of women being part of this intervention (Kurtiş et al., 2016). While the relational embeddedness of women and their impact on empowerment has been studied by various scholars (see Huis et al., 2017, 2020 for some examples) we focus in this paper on the dynamics of the differences in power distance between the facilitator and the receiving community and its impact on the empowerment intervention, in a cultural setting characterized by tight norms and strict gender roles.

### Power Distance

Power distance refers to the degree to which individuals, groups, or societies accept inequalities in power as unavoidable, legitimate, or functional (Hofstede, 1984). Power is present in every relationship and the acceptance of inequality in power influences how individuals and groups with different degrees of power will interact (Daniels and Greguras, 2014). Therefore, power distance shapes the perception of justice, the link between attitudes and behavior, the types of elicited emotions, as well as leadership expectations (Daniels and Greguras, 2014).

For instance, individuals with power are expected to lead autocratically. These expectations can even cause ambiguity or suspicion in high power distance followers if leaders take on a more participatory approach (Kirkman et al., 2009). This is because those with less power have a greater need for rules, directions and instructions, for which they prefer to rely on their leaders (Hofstede, 1984). In turn, figures with more power should be respected and shown due deference (Yang et al., 2007), and therefore people low in power behave generally in a submissive, loyal, and obedient manner toward their leaders (Bochner and Hesketh, 1994).

The interaction between people at different ends of the hierarchical ladder is tilted, in the sense that those with less power will be more lenient (Ng et al., 2011), positive (Varela and Premeaux, 2008), and respectful (Bond et al., 1985) in their upward feedback in order not to threaten social order and the power hierarchy (Daniels and Greguras, 2014). At the same time, individuals with less power feel more hesitant to initiate interactions with their leaders and to solicit their feedback (Daniels and Greguras, 2014). Feedback among peers, having the same level of power, seems more accepted and comfortable for both individuals low and high in power (Hwang and Francesco, 2010; Daniels and Greguras, 2014).

In terms of compliance or challenging existing norms, individuals with less power are more willing to mimic the behavior (and judgment) of their leaders and show outer compliance. This, however, does not automatically imply attitudinal conversion (Daniels and Greguras, 2014). This outer compliance is related to the fact that when challenging the existing social hierarchy and norms, people low in power are more likely to experience violence (Daniels and Greguras, 2014). Furthermore, when aiming to alter power relationships in their advantage, they run a risk that can be materialized in the public or private sphere. Recent research shows that in such cultures, those who challenge the norms are seen as less powerful and are often ostracized because of this (Stamkou et al., 2019).

Given these arguments, we conclude that the behavior of individuals will depend on where they situate themselves in terms of power in relation to others and to what extent they value power distance. An individual that is low in power and rates power distance high might not see the need to challenge inequalities. Even if this person has ambiguous feelings about power distance and perceives it as unjust, it does not necessarily mean he/she will take action to challenge it because of the possible negative repercussions – either initiated by those with more power, e.g., violence or the denial of access to vital resources, or ostracization by the community for disrespecting social norms (Pratto and Walker, 2004; Kabeer, 2011; Stamkou et al., 2019).

## Methodology and Research Context

In the current study, we research such a process, where a participatory method was used in a high power distance country (i.e., rural community in SNNPR in Ethiopia) to foster women empowerment in agriculture (i.e., rural settings). This participatory process was part of the “The Gender and Youth Empowerment in Horticulture Markets (GYEM) project. GYEM was a Comic Relief-funded project running from February 2016 till June 2019 in Ethiopia. SNV Netherlands Development Organisation implemented the project in two regions, Oromia and SNNPR, together with two local horticulture farmers’ unions and the local government. The overall goal of the project was to ‘enhance women’s and youth’s social and economic empowerment through improved access to and control over assets and benefits in the horticulture value chain.’ To that end, five result areas were defined, of which the last one was: The communities, unions and cooperatives promote women participation and decision-making power in horticulture value chains.

For this result area, the methodology PALS was selected in order to work on gender equality and power imbalances between men and women. The PALS methodology, while developed in collaboration with NGOs operating in the Global South, is nonetheless anchored in an individualistic, neoliberal view of gender equality and empowerment. This is reflected in some of the basic assumptions of the methodology and the resulting tools. Appendix 1 summarizes the principles and assumptions of the PALS methodology. The two main parties in this process started from large differences in the power distance dimension: the Dutch-originating NGO which provided the intervention was low on the scale (Netherlands scoring 38 on power distance according to Hofstede) and the beneficiary, an Ethiopian community, was high (Ethiopia scoring 70 on the same dimension – Hofstede, 2020).

We explore how these differences influenced the effectiveness of the empowerment intervention based on the PALS methodology through an adaptation of a framework developed for assessing cross-sector partnerships (van Tulder et al., 2016). We consider this to be a cross-sector partnership because the intervention involved farmer’s unions, cooperatives, an NGO, community members, and kebele (village) administrators. In his model, van Tulder et al. (2016) proposes that to assess the success of such a partnership, one needs to look not just at the end result, but also at the process leading to this result. The model allows for a detailed analysis of the differences in values, assumptions, and interpretations of the parties involved and looks at the matches and mismatches regarding mission, input, throughput, output, outcome, and impact. We use a simplified version of this frame and focus the analysis on mission, process (what van Tulder calls throughput), outcomes (i.e., the immediate results), and impact (i.e., the longer-term effects and ripples). We differentiate between two types of effects (i.e., immediate and focused effects which we call outcomes and longer-term and rippled effects which we call impact) to add the social change dimension to our analysis. By looking at longer-term and broader impact, insights regarding the sustainability of a partnership, and a more in-depth analysis of the reasons why a partnership fails or succeeds, come to the foreground.

After describing each component of the model, we present our findings within this frame.

### Data Collection

Both primary and secondary data were used for the assessment. The primary data consisted firstly of 42 interviews: NGO and GYEM project staff (seven men and five women); two union board members (two women), five women champions^6^ (i.e., participants in the original training), two wives of men champions, two women neighbors and 19 second round champions (i.e., villagers trained by the original champions – 8 women and 11 men). The interviews were conducted by five people: the two authors of this paper (two women of which one was the project manager of the project) and three research assistants (two men and one woman), all Caucasian European. The language used was Amharic, and to this end, the interviewers were assisted by Ethiopian translators (two men and one woman). We tried as much as possible to have the woman Ethiopian translator cover the interviews with women, but this was not always possible (a third of these interviews were translated by men). The male Ethiopian translators were field project staff who had been facilitating the empowerment process through PALS and knew the respondents well. Their interviews were then transcribed and re-translated by an external woman translator, to check that the translation was accurate and unbiased. The interviews asked interviewees for their life story chronology, asked to describe a normal day in their lives, inquired about their family relations, people they respect in the community, what makes a good woman, and what they hope for the future.

Secondly, we used an assessment checklist (excel) for the 20 champions that were originally trained in PALS and that were filled in, based on semi-structured interviews, by the (male) field project staff. These interviews aimed to understand the economic, social, and personal changes that champions made in their lives as a consequence of the PALS workshops, as well as assessed their sources of power, such as age, wealth (of original family and current family) and level of education. In a second phase, 1 year later, we discussed changes that were sustained over time and changes that did not take hold and, for the latter, possible explanations for the failure of the change to become an established form of praxis. Furthermore, changes at the level of the champion’s partner were also discussed in the second interview, as well as possible negative effects of the intervention.

Furthermore, there were two ethnographic observation episodes (each lasting a week) with three women champions. The authors of the paper lived with various champions for the duration, gathering extensive information about their family dynamics, daily routines, and social dynamics in the village.

The secondary data (following the procedure proposed by Gray, 2008) originate from the reports of staff members (mostly the project manager, who is the main author of this paper) during observation of trainings and follow-up conversations with different champions and their peers. Fifteen field observation episodes during workshops and follow-up field visits were used to inform the data collection of the primary data and to provide background for the phenomena observed, like for instance men coming late during workshops or interrupting women during workshops.

Following Bhopal (2010), we acknowledge the complexity of the relationships between the researchers and the research participants in this setting. This complexity is situated at multiple levels. The Caucasian researchers can be considered as outsiders by participants and their nationality, race and class inevitably induced difficulties in understanding the ‘other’ (Bhopal, 2010). To compensate for this bias, we, as principal researchers, aimed “to capture the ways in which multiple forms of oppression impact women’s lives and empower then to tell their stories by providing a respectful and egalitarian research environment” (Campbell and Wasco, 2000, p. 787). To this end we spent several days in the community, after having requested official approval from the local authorities, and participated in the daily activities of the women. Our gender also proved to contribute to a shared identity (Bhopal, 2010) and facilitated the research. This can be illustrated by the fact that the rural community adopted strict gender rules where some members of the community refused to shake hands with people from the opposite sex [one of the interviewees Z. apologized for not shaking the hand of our male colleagues because it was not allowed by her (Muslim) religion]. One instance highlighted that an outsider status can shed light on issues that are taken for granted by insiders (Bhopal, 2008). The project manager noticed that the eldest girl, aged 14, of the family she resided with (B) did not go to school during the day. When inquiring about this, the Ethiopian female translator acknowledged that she had not noticed it since it this is a familiar situation to her.

At the same time the translators, from Ethiopian origin, can be considered insiders to the research context. They did not merely translate but also adapted questions to the local reality of the interviewed and provided additional information were needed. They also administered the assessment checklists. Although ethnic matching is advocated by various researchers (Ashworth, 1986; Kauffman, 1994; Papadopoulous and Lee, 2002; Bhopal, 2009), there are still differences such as status and gender to be considered in relation to the male Ethiopian field agents that could have increased the distance between them and participants and impacted the results. Through the translated transcripts for instance, we discovered that in few instances the field agents had not translated gender-related sensitive issues for us. As described below, we tried to be conscious of the different biases related to power and culture that impacted the multiple relationships between the researchers, translators and respondents. We acknowledge, however, that our race, gender and status might have hindered the process and the desirability of the answers.

### Data Analysis

The analysis and collection of data followed the principles of thick description (Geertz, 1973), which is defined as the process of paying attention to contextual detail in observing and interpreting social meaning when conducting qualitative research. This was done in a feedback-loop process, with analysis informing further collection, which spurred new analyses. The steps consistently taken throughout the analysis were the following: First, we decided which data sources and techniques for collection we needed for each stage of the van Tulder model. Different sources of data were used to assess each stage of the van Tulder frame, according to their content: the workshops observation reports and interviews for inputs, throughputs and outputs; the assessment checklist and interviews with champions were used for outputs and outcomes. The interviews were coded in Atlas.ti, using a combination of grounded and theory-informed coding. The codes covered both individual-level aspects (e.g., aspirations, economic activities of the person in the household, economic improvements, social capital, values), group-level (economic activities of the household, power relations in the village and within families) and cultural-level ones (such as gender norms and values, indicators of tightness in the culture). The assessment checklist was analyzed manually by counting the various categories reported for each type of change (e.g., economic, social, personal).

Second, we combined the observations noted during workshops, field visits and interviews (i.e., qualitative techniques) with reported changes (quantitative assessment) and quantifiable assessments of tools understanding and usage to derive hypotheses and questions. These hypotheses and questions were then explored further in the interviews or by purposefully including them in elements to be observed during following field visits.

Third, we modified follow-up data collection to pursue these questions and hypotheses. For instance, the assessment checklist was produced after several observations and interview episodes, and it expanded as new phenomena were observed – such as non-intended negative consequences, which had not been covered at first.

Finally, as we found strong evidence building toward a certain conclusion, we challenged that in our internal discussions (i.e., the authors had bi-weekly calls) using the devil advocate’s technique and intentionally searching for disproving evidence or alternative explanations. Furthermore, we used three ways of triangulating the data before drawing our conclusions.

Triangulation was used to increase the reliability of our findings in three ways: (1) triangulation between different data sources (e.g., assessment with interviews; workshop reports with follow-up field visits); (2) double-checking the findings and interpretations with local Ethiopian NGO employees (who had no stake in findings going one way or the other); (3) double-checking our findings and interpretations through two final interviews of expert local researchers with experience in research in gender and agriculture in Ethiopia. The data-source triangulation meant that we first processed the data according to their original form (i.e., quantitative analyses for the excel sheets monitoring; coding for interviews, and for the activities and events described in reports). Based on this level of analysis we proposed a first set of findings. We then selected a finding as relevant if it was present in more than one source (so both in field observations and reports or in monitoring sheets). We looked for common patterns and consistency in the patterns. We then double-checked our interpretation of those findings through the interviews with local staff and/or with experts. Often these interviews offered new views or interpretations. When that was the case, we went back to the data and sought the evidence for the newly proposed interpretation. If we could find evidence in more than one data source, we integrated it in our story.

Finally, we opted to report our findings using thick description, so that the reader can actively think through our findings through the lens of the context they emerged from. As Geertz (1973) proposed, we see and understand in contexts, “as we conjure our interpretations of what is going on. It is only by allowing ourselves to be guided by the entity of study and critically questioning the complexity of our contextualized responses that we can gain a better grasp of this complex architecture that is analysis” (Freeman, 2014, p. 287). In so doing, we hope to make the reader part of the conversation and to spur their interest to further explore some of the insights we propose in this paper.

## Findings and Discussion

### Cultural Context

In addition to relying on previous country-level research that characterizes Ethiopia as a tight (Gelfand, 2019) and high power-distance culture (Hofstede, 2020), we triangulated the statistical data source with insights from our interviews. The tightness of a culture is indicated by backlash to norm challenging and the resistance of a system to change. In our interviews, this was reflected in topics around challenging social customs and the price paid for it– (What do the others say if you miss out the coffee ceremonies?) “They would think that I am being proud. I am not that much into coffee ceremonies. I want to wake up in the morning and prepare food for my children. Then I work out in the field. I bring food to the cattle. While I do this I miss coffee with others and they say to me that I am being proud and keeping myself busy.” (B), as well as women’s relatively set place in the community and their dependence on marriage: “I try to accept that it is what I am; since we have kid together, even if I leave him, I would get back, I would not get better husband” (Ze); “What good is a home without a husband? Your children even want to rob you…the children want to take their share of the land…when I face such things…when my sons wish my death in order to divide the land, I bring my brothers home to reprimand my sons. They would tell them “if you ever bother her, we would report you to the police.” Then my sons stop bothering me. Even Hassan—it was like yesterday when he was a small boy – bothers me about land now. He wants his share. Unless I give him some benefit, he won’t obey me. I even pay the monthly membership contribution for Iddir –I give him 50 birr. There is nothing for free” (Be). “He didn’t [send elderly men to ask her for marriage]. I told her [the niece] to stop the relationship. He is not someone we chose. We forced her to stop seeing him. We made her break her relationship with the man. She is now living in a way we want her” (B). This latter topic also relates to women’s lower place in the high power distance system. Furthermore, the high power distance is indicated by deference to others based on their age, gender or status: “I respect them [n.n., elder people and family] because they are my parents. I am younger, I have to serve them” (S); “The imam teaches us to respect our husband. He doesn’t specifically talk about good woman or good man but generally tells us to respect our husband” (M).

Furthermore, an interesting aspect regarding women’s place in this society, was how women themselves keep the *status quo* while seeing the benefits of change. Specifically, while all mothers we interviewed acknowledge the importance of education and see it as a means to a better life (“I teach her that if she is educated, she will become successful. If married early, I also tell her the drawbacks. I tell her to prioritize her education” – H), they also object to the norm breaking that comes with the younger generation: “At the Woreda level, I represent the kebele’s Women’s association. I am highly concerned about girls’ education. I don’t want girls to drop out of school and also tell their parents not to encourage such things. Even if I can’t afford to buy education materials like exercise book and pen for them, I push forward the issue of girl’s education and spread the idea that girls will benefit the country if educated. I fought against harmful traditional practices. In one instance I was even beaten for siding with an abducted woman. I filed a case against the abduction. The girl was set free, thanks to God she is now attending 7th grade. Women’s affair covers wide ranging issues…such as “cutting” [n.n., genital mutilation] and abduction…dropping out of school…I am one of the activists pushing for the eradication of such practices. I consider all women as if they are my daughters and sisters. (What do you advise young girls?) I advise them not to have many friends…stay out late… (Do girls stay out late?) Of course they do…they usually come up with a pretext to bring book from friends …they have such kind of lame excuses…you know.” (A); “my time was much better than their time…now girls don’t care about family, they can talk to anyone, get married to anyone, they don’t give respect to us. My time was much better than theirs, we were reserved, we didn’t just speak to anyone on the street” (Ze). At the same time, they perpetuate the ideal of a good woman as an ‘eshi’ (i.e., yes)-woman: “A good wife is someone who keeps the house clean, herself clean and welcomes her husband when he comes home.” (Z); “they [n.n., the wives] have to obey. The man bought the wife…he paid money for her mother…therefore she has to obey. Hasn’t she!?” (Be); “The woman has to have medical test. She should be able to work in the house. She should have uniform stand and should not go here and there with other people. Should prioritize her household… She should prioritize her husband and children” (G); “A good wife is someone who manages to go to the market and provides for her children. She raises her children very well. We get our profit from the market. If she manages to earn well then she is good. In this village, a hard worker who manages to get profit from her sales is a good woman. If she stays in the household, she will spend her day in the house and with the children” (H).

We have in these illustrations the picture of a society with a tight normative system, where women’s roles and gender norms are very traditional – and upheld by most members of the community. We will see throughout the findings on which dimensions it is challenging to support gender empowerment in such a society, how power distance moderates interventions in this direction, but also how understanding such a system allows to use the existing constraints to boost empowerment.

### Mission

The mission, being the first component in any cross-sector partnership: is the reason for the respective partners to enter a partnership. We contrast PALS’s mission with the mission of the community. The mission of PALS is to empower communities through enhancing control over one’s life and catalyze a sustainable movement for social justice (Mayoux, 2018). The content of social justice, i.e., what it means to the facilitator and what it means to the community, is not made explicit throughout the process. This is to ensure that people are themselves responsible for the content and process of the methodology applied to reach sustainability. Gender equality is, in this methodology, an implicit goal of PALS, as gender is mainstreamed throughout and is considered to increase the effectiveness and sustainability of the intervention (Mayoux, 2010, 2018).

The mission of the community, however, has primarily an economic focus, rather than social justice or promoting gender equality. We especially observed this in our interviews while asking two sets of questions: what would make the women happy and what would they deem important to teach their daughters. For the first question, the answers were along the following lines: “If I can afford the motor [i.e., water pump – which was given by the NGO], I want to grow onion and carrot […] My plan is to get the motor and work. Get the motor and work…! That is what I want and what makes me happy!” (Be); “I want my children to grow up and be happy […] if I provide them with food that is necessary for their health, school bags, things they need to learn, then they will be happy I think” (S); “A better life, I want to look good and clean. And I want my children to live in the city. […] In the city they would have a chance to do business. In the rural areas, there is a shortage of land and electricity.” (Ze). For the second question, the answers tapped again into the role of financial security in a happy marriage: “Explain [n.n. to my daughter] about marriage, what makes it miserable […] if you don’t have money to give food, education etc, then what’s the point of having children, being married. […] there is nothing good about being married. Because we are poor, I’m saying that marriage is misery; but every time I had a new kid, I have reason to live; but it’s the financial the issue” (Ze); “They [n.n., husband and wife] would be happy if they are financially secure. A husband and a wife usually argue over money matters. […] If you have money at hand you will always be happy” (M).

As was reflected in the interviews held with champions and their peers and observed during the ethnographic field stays, most people in rural Ethiopia struggle to make ends meet, inevitably leading to a concern for money. Consequently, the primacy of survival values over self-expression is characteristic to low-income countries, in Welzel et al. (2003) taxonomy. 60% of the first-round champions ascribed the support they received from their network to share the PALS tools to the learning peers could acquire about effective management of income and expenses. Furthermore, the motivation to listen was underpinned with the desire to increase their material living conditions, as champions confirm in the interviews: B. shared that members of her community repeatedly accosted her to inquire about “what tangible things she had received.” Likewise, her son questioned the utility of the training, “if she did not receive the water pump she wants.” The concern for money and material resources appears to prevail over other topics. As one champion stated: “People did not have a clue about their expenses and income. During the trainings they came to the conclusion that they have to reduce their expenses.” These quotes were repeated in various forms by other champions and their peers and toward the end of the project, some even reported that the training was intended to get rid of addiction and unnecessary expenses.

One year after the intervention, the economic benefits remained the most important take-away of PALS: 80% of the original champions considered the reduction of costs as their biggest accomplishment. Changing gender dynamics and increasing social justice were barely mentioned, despite being the intended result of the methodology. Where an increased level of understanding between husband and wife was reported (i.e., by half of the champions), this was explained by more transparency about and better management of their respective expenses and sources of income.

### Throughput

Throughput looks at the activities where inputs are used leading to the desired outputs. This mainly concerns the organization of the PALS trainings according to principles like facilitation from the back, the organization of community open days, the individual monitoring by staff in between, and the sharing of the original champions with their peers. In high power distance contexts, participatory methodologies can conflict with less powerful people’s comfort to reflect and express their ideas in public. Therefore, for the scope of this paper we focus on facilitation from the back as a core principle during the trainings and all PALS-related events.

#### Facilitation From the Back in a High Power Distance Context

Facilitation from the back is based on the assumption that change comes by giving equal voice to all people who are part of the process. Consequently, the method is designed to challenge existing power inequalities by everybody participating in an equal way. The principle was key during all trainings and monitored through for instance measuring how long or how frequently one participant would speak.

An external consultant led the workshops and in parallel trained the local (i.e., Ethiopian) NGO staff in the methodology. Because of the consultant coming in as an expert and thus higher in hierarchy, she/he had ‘the power’ to challenge the perceived power dynamics and to facilitate from the back. However, local staff, who were responsible for closely guiding the trajectory in between workshops, didn’t have the same degree of authority and power and thus could challenge the farmers to a far lesser extent during the process. Furthermore, from the interviews with the staff and observations of their interactions during the workshops it resulted that in most cases they didn’t want to challenge hierarchies, as they too – coming from the same culture as the farmers – valued more the top-down transmission of knowledge, thereby reinforcing existing power hierarchies. This issue was misinterpreted by the consultant, who repeatedly raised the issue under the assumption that the local staff “didn’t understand the methodology.” Our follow-up interviews reveal that the divide goes deeper than not understanding the methods, into this approach simply being incongruent with their world views and values, and an external imposition on their cultural practices and challenge of said values. This caused local staff and farmers to often act against the facilitation from the back principle by telling others what to do instead of inviting participants to share their ideas and having long unidirectional plenary sessions. This was observed repeatedly during various field visits. One incident stood out where a government official, clearly higher in power than the farmers, was ordering the champions what to do, how to draw and even completely erased a drawing of a champion in front of the whole audience (June 2018, upscaling Soddo). This dynamic was not understood by the consultant, and because the reporting of the throughput was done also within their indicators of success, this was reported as a lack of comprehension or compliance on the part of the community, instead of what Foucault calls a ‘micro-resistance’ toward cultural impositions.

In addition, the external facilitator can only perceive power imbalances from her/his own culturally influenced perspective and might misunderstand or misinterpret a particular event as a sign of power imbalance and revert to facilitation from the back in order to supposedly correct the situation. For instance, a woman not talking in front of a man might be perceived as a woman being overruled by that man, even if she wanted to speak up (Jackson, 2012). During field visits and workshops, we observed this phenomenon multiple times. However, in small groups, women who were assigned as trainers, often did speak up and guided their trainees, including men, in their drawings. This was not necessarily a consequence of facilitation from the back but rather of the power status held, given that the woman was a trainer, in that specific situation. The interpretation of when facilitation from the back is appropriate to reach the intended result is thus very personal and bound to a particular context. As such, identifying and respecting cultural preconceptions and a hermeneutic approach toward contexts is clearly vital for laying the groundwork for a successful mission.

### Outcomes

In this section we describe the positive effects observed and mentioned by champions as a result of participating in or being influenced by the workshops. The effects were recorded in spring 2018, one and a half years after the first workshop.

#### Economic Outcomes

More than 60% of the champions mentioned the creation of an individual source of income and the diversification of earnings as a positive outcome. Fifty-five percent of the women invested in cattle, in particular poultry and livestock (sheep), which are typical female assets. These findings from the individual interviews correspond to the follow-up assessments where 50% of the women stated to have invested in some kind of asset and 100% of the men had made such an investment. The intervention clearly opened up the possibility for women to be more efficient in their gender role but did not necessarily challenge the power relationships along the lines desired by the intervention and within the definition of empowerment held by the Western-based intervention providers (Kabeer, 1999). As previously stated, due to high power distance, women (and men) might not see the need to tackle women’s gendered roles resulting from their low power as women, but women did seek to become more efficient and effective in those roles. This is in line with previous critiques of Western-based empowerment interventions, which “might provide a well-positioned minority with the resources to better operate within existing power structures” but “tend to reproduce systems of oppression” (Kurtiş et al., 2016, p. 390).

One very important aspect to note here is that, even when it comes to the economic outcomes reported by the champions, quantitative methodologies (used to measure outcomes according to the criteria necessary for donor reports) are highly biased in high power distance communities, such as rural SNNPR in Ethiopia, again, due to the tendency for socially desirable answers. Compounded with the desire for improving their material conditions, answers are tilted toward the positive not only to please the facilitator but also to invite the donor provider to continue the support. This observation resulted from the discrepancy in some of the reported data with observations in the field and inconsistencies in interviews and was confirmed through the interviews with two gender experts with decades of experience in international development work in Africa. Both experts cautioned in the types of measures most projects’ use for assessment and pointed to the need for more in-depth, qualitative insights in assessing outcomes of projects. The field agents used as translators in some of the interviews were also contributing to creating a more positive image of the situation. For instance, when asked how people react to the tools when she shares them, champion G responded “They are happy. Recently we are planning to undertake some activity and we have drawn out some money. They are eager to work and save money” whereas the field agent translated “They are very happy. Especially about the advice on saving. I am starting different kind of activities on the vision…horticultures… she has head cabbage, leaf cabbage and khat…she has started selling these things. She is experienced after the training and the others heard about her. The VSLA^7^ members even borrow money from the VSLA. They have started taking loan and she has also taken that loan. The reaction is very good. They listen to us attentively. Once they are trained, they should share it to the others. The community knows her progress and they are very happy.”

#### Social Outcomes

Fifty percent of the women champions mentioned the increased respect of the community and being listened to more as an improvement. It is thereby striking that 90% of the original champions mentioned this as a positive effect, while no second-round champions did so. It is not only an increase in knowledge which increases respect within the community, but also having been trained by an international consultant instead of a peer. The training by the consultant was closed with an Open Community Day, a festive ceremony with the broader community, during which the champions were certified. Through this certificate, champions increased their credibility as a trainer, as illustrated by following quote: “During her sharing she also showed the certificate to them. It created trust that she is considered as trained and capacitated” (S). Furthermore, during the assessment, one woman champion testified that “Since she was trainer and sharing the tools, her change areas, and experience to the community, they are listening to her.” Being able to devise a plan toward material betterment remains a clear path to gaining credibility; not just within families and communities, but also within formal social relations, i.e., with government authorities. Government officials were overall interested and impressed by the plans presented in the follow-up meetings, despite declaring that they would not have imagined farmers capable to have a plan. This shows the two-way dynamic of credibility and status: not only does status confer credibility, but newly proven credibility can confer status. In this, we see also a possible route for social change.

Remarkably, only 20% of the men champions mentioned increased respect as a positive outcome. The training and/or the changes they made were thus less perceived as contributing to their status compared to women.

We see different possible explanations for this differential outcome. First, the leap for women was greater since they often start off with less education, less knowledge, and less visibility. This methodology not only increased knowledge of tools but also required women to share the tools through which they could demonstrate their knowledge and help others in making plans for change. We infer this outcome might have helped them to climb up the hierarchical ladder, yielding more power and thus more status, as long as the sharing and changing continued. At the same time, the changes men made were mostly about chewing less chat and drinking less alcohol, which is a regular group activity among friends. Some male champions reported they lost friends over it. Although sometimes they could influence their friends to also cut back on chat and alcohol, they might have lost influence and status in relation to their peers, because of less social gatherings. Another possible explanation is that men traditionally occupied more social positions within the community compared to women. Before the intervention four men occupied a leadership position (as a chairperson or leader) of a formal (i.e., kebele council) or informal (i.e., edir) structure opposed to one woman. After the intervention, however, five women and two men reported to have moved up the hierarchical ladder toward a leadership or board position in one of those structures. Moreover, 70% of the women joined such a structure as a member. Starting off with a lower status position, leaves more opportunities for climbing the hierarchical ladder and it seems that most women seized this opportunity as well.

Over 40% of the participants reported that relationships at home had improved because of better mutual communication, consultation, and discussion. During the first assessment, this was reported by 75% of the original champions as the biggest success story. The assessment shows that this discussion is mostly about economics, expenses and savings. Kabeer’s (2011) study of women empowerment in Bangladesh points to the same pattern: women gain more access to education (i.e., their husbands allow them to attend trainings) and more weight in household decisions when they can prove that these trainings result in material betterments for the family. As G, another champion, reports: “Back then he was working on tomato gardens. I told him what I learned about vision and informed him to plan and work according to his plan. I also shared my vision journey. I told him that we should work by planning and explained that the tomato garden he was working on should be done by plan. I told him about my plan and he was surprised to see it. He accepted my plan.” Several champions show a similar shift in power relations within the household after the training, because the women participate in concrete discussions about financial gains for the family due to the tools they learnt. Our assessment revealed that 80% of the women and 70% of the men reported more discussion in the household about economics, saving etc. as the biggest change. However, the different missions of partners are reflected in the different success criteria of the project: the facilitator was looking for more gender equal outcomes, while the economic outcomes were most prevalent and most mentioned by the champions themselves. As the results show, more than half of the participants that did not mention the improved relationship during the interviews. Moreover, some second-round champions seemed to have no idea that the training was about gender. People mostly picked up the economic changes: increasing income, cutting expenses, and to a lesser extent increased intra-household decision making.

### Impact

Impact encompasses the ultimate, mostly long-term direct and indirect effects of the partnership on society. The effectiveness of the partnership is expressed by assessing to what extent it has improved the issue, in this case increased gender equality through disseminating a shared gender agenda which enables women to enhance their participation in the horticulture value chain. We split the impact between dissemination of the methodology through upscaling and the perceived indications toward women empowerment and gender equality.

#### Upscaling

To upscale the methodology, selected first and second-round champions trained new champions in other districts. This process was initiated about 1 year after the original trainings and applied the same selection criteria, workshop set-up and facilitation principles as the training by the consultant. These upscaling trainings, replications of the original training, confirmed how difficult it was for farmer trainers to facilitate from the back. Instead, trainers displayed their knowledge by talking a lot and explaining not only the tools but also providing content to the tools. This led several farmer participants to simply copy the procedure without going through their own process of reflection on their future plans and potential changes. Social learning theory (Bandura and Walters, 1977) points to learning from role models one identifies with, through observation and imitation as a core learning tool. Imitation is organized cognitively in a hierarchical fashion (Byrne and Russon, 1998): from simply imitating content to process models. The habit of peers who were in training to copy the drawings of the champion-trainers signals a desire to mimic these successful/esteemed members of the community. This is typical for high power distance contexts, where social reproduction ripples down the hierarchy as a core social learning tool. In such a setting, facilitation from the back comes then against the core of this learning process. In this phase, our findings show that the facilitation from the back principle was not observed at all.

Looking at our findings through this lens, we see the value of the learning process, despite the core principles of participative methodologies being violated. Farmers selected to train their peers – who therefore enjoyed higher status and authority – took over the facilitation process. This was especially the case in very public settings, with a larger group, where men would take the floor during the upscaling training and talk lengthy with lots of gestures. In those settings, women spoke quietly, briefly and were easily ‘chased away’ by men when they said something (perceived as) incorrect. Within small groups however, as opposed to plenary sessions, women were more frequently taking the lead of the training process of their peers. This points to niches where change can happen, not in opposition to local customs and hierarchies, but in a complementary fashion.

#### Gender Equality and Women Empowerment

Leaving questions of social justice and the gender agenda open-ended avoids the potential risk of participants copying ideas from the facilitator and gives the opportunity for the community to define their own empowerment process and outcome. We regard the open agenda as crucial to ensure that one form of subordination (women versus men) is not replaced by another (community versus facilitator). On the other hand, leaving this agenda implicit, compounded with power distance leading the community to incline toward external compliance with the presumed gender agenda of the facilitator, might reduce any opportunity for the community to reflect and act upon their own shared understanding of gender equality.

Some behavioral and attitudinal changes can be translated into a tangible impact if the effect is considered in the long-term (i.e., cross-generational) instead of immediate impact. This is a pattern that transpired in all our interviews with women: different aspirations for their daughters and higher investment in their education, alongside with changes in patterns for marriage pressure. For instance, champion H declared: “I teach her that if she is educated, she will become successful. If married early, I also tell her the drawbacks. I tell her to prioritize her education. […] We were not encouraged to learn. Now education is their right. Women will not get married if they don’t want to. If possible, I want to work hard and educate my children further. […] We encourage children to go to school. Many parents let their children work in the household after they came back from school. But I don’t let my children to work after school. I am not saying that working is bad however too much work will adversely affect education.” Along the same lines, champion S is sending one of her daughters to school in another town and is able to pay for it from her increased savings. Champion A depicts the larger pictures similarly: “At this point in time, even at the country level, such practice [forced marriage] hardly exists. These days the practice is totally different. Girls attend schools at the time they are 7 years old. Back in the old days, school girls were bothered by early marriage. Even students were not guided by plan and their vision was narrow. As compared to this situation, there is a big change.”

Many methodologies fall into the pitfall of the Western short timeframe of implementation and assessment (use Western-based conceptualizations of gender equality). As such, they look at gender empowerment within a generation, and even more specifically, at the level of changes in the behaviors and attitudes of participants in the program. Because of that, they miss out on attitudinal changes that flow to the next generation. In our interviews, all the women hoped for themselves was material and immediate. But when asked about hopes for the future, a desire for change transpires: “A better life, I want to look good and clean. And I want my children to live in the city. In the city they would have a chance to do business. In the rural areas, there is a shortage of land and electricity” (Ze). It is for the next generation where a hope for a significant leap in the quality of life (and active investments in it, such as women capitalizing on their children’s education and desire for them to become (or marry) civil servants – a high status, stable income job).

There is a bias toward reporting quantifiable and immediately visible results, whereas the real impact in traditional (i.e., tight), high power distance countries, as we see here, happens slower in time, and often through the mechanisms of changed aspirations for future generations. Here is where we observe that PALS leaving the gender agenda open is actually an asset of the methodology, allowing participants to conceptualize and approach the issue in their own way and at a pace compatible with norm changes in tight societies. Though it seems that men and women did not actively pursue changes related to gender, women did express a desire for their daughters to have a different life. This is something the methodology could have tapped further into – as mentioned in the mission – to help women express more clearly how such a life would look and what would be necessary to achieve it. In building hopes for their daughters, they start imagining different lives – and these images become part of the collective representations of gender and tools for gender norms changes.

## Limitations of the Study

The researchers of this study were both Caucasian and European. Being external to the Ethiopian context meant the researchers brought their own assumptions and possible misunderstandings to what they observed, or even what they observed. We tried to counterbalance this in several ways: first, one of the researcher is born in an Orthodox, tight-culture country; the other has over a decade-long work experience in several countries in Africa. This allowed us to perpetually and actively challenge each other’s assumptions about what we saw, and share observations and interpretations. The different mindsets helped to not always see the same thing in the field, and question our observations. To help with the emic understanding, we relied on triangulation interviews with local Ethiopian staff (both from the project but also outside the project), and experts – gender researchers with experience in Ethiopia (one local and one with international experience in Global South research).

Furthermore, the externality of the researchers also played a role in the power dynamics with the local community. On the one hand, this led respondents to provide a number of socially desirable answers (especially when it came to changes stemming from the training, when asked by the project manager). This was not only observed vis-à-vis the researchers but also in front of other people higher in power. For instance, when B. was asked during an ethnographic field observation, whether she preferred the PALS methodology or the VSLA groups, she spontaneously replied VSLA. However, her husband corrected her and encouraged her to choose PALS over VSLA, which she immediately, without further discussion, did. Hence, the answers of the respondents might not always correspond with their personal opinion. On the other hand, this strengthens our assumption that in high power distance settings people show outer compliance, which is a possible pitfall to participatory methodologies.

Because of having to use translators, we missed some nuances and a natural flow in the interviews. Translators might tend to simplify the response or might also leave out sensitive information. We noticed this with the male translators (staff), when double-checking the translations in writing (we asked a second translator to transcribe and translate the full recording, signaling separately what the interviewee said and what the translator told us). Because of the discrepancy between what the interviewee said and the translated answer (which was occasionally biased toward social desirability – both to acquiesce the success of the project but also to make the community look good in the eyes of the researchers), we were not always able to have a proper conversional flow, or to go in-depth with certain strands of the conversation. As soon as we saw this in the transcribed interviews, we decided to hire a female translator for the ethnographic field visits. Moreover, the project manager had learnt some Amharic and could detect when some parts were not translated.

One aspect that could be seen as a liability for our study is that one of the authors was the project manager of the intervention. To counteract for the limitations her status would bring to the dynamic with the participants, the project manager was involved in field observations where she stayed several days in the community in order to build trust, with the aim to ease the evaluative atmosphere. She also clearly expressed during the field stay that she was there to learn and to understand the situation. The fact that the project manager was known by the community (she had done field visits on a regular basis before) and had always asked for advice from the community instead of telling them what to do, facilitated this process. While we certainly acknowledge the evaluative angle in the dynamic with the participants, we also want to stress the counterforces put in place to compensate this angle and help make the interaction more complex and nuanced.

## Conclusion

The PALS methodology created a great deal of enthusiasm among the participants who were part of the workshop, along with staff and representatives of the government. The peer sharing, when it occurred, proved to be very inspirational and motivational. At the heart of the success of the methodology was the reliance on people’s personal reflection and internal locus of control. Possibilities opened up that were unknown to participants before. This adds to the emergence of real choice, because women can now envision more pathways for themselves (Kabeer, 2017). However, our main point is that cultural differences between intervention designers and facilitators and local communities need to be acknowledged, reflected upon and worked with, in order to maximize the impact of these interventions long-term. This advocates against a “one-size-fits-all” approach and a thorough investigation of and reflexivity on cultural dimensions of how women in different parts of the world experience empowerment (Cornwall, 2003; Mohanty, 2003). Specifically in the case of differences in power distance, if participatory methods deny the power dynamics in place or appear to judge them because of the intention to change them, these dynamics will surface anyhow, as we clearly saw during the upscaling trainings and reduce the impact of the methodology. Adjustments need to be made for these methodologies from their design principles to the selection of participants and implementation, as well as in the way outcomes and impact are being assessed. More contextualized research, some done by researchers external to the project, but some done through active reflection of the facilitators, and sharing of the insights gained (not just the success stories) are necessary for progress in the field of international development interventions. We hope, through our storytelling of this case and the recommendations suggested as a result in Appendix 2, to have contributed to this global conversation.

## Data Availability Statement

The datasets generated for this study will not be made publicly available part qualitative research and part reports that belong to SNV.

## Ethics Statement

The studies involving human participants were reviewed and approved by SNV Ethiopia. Written informed consent for participation was not required for this study in accordance with the national legislation and the institutional requirements.

## Author Contributions

SB was in the lead for the design of the research and its theoretical grounding, and participated together with SDS in the ethnographic observation and interviewing process. SDS was in charge of the data collection process, collected a large part of the data herself (across all methodologies), and was in the lead for writing the manuscript, with multiple reiterations between both authors. Both authors contributed to shaping the direction of the manuscript and to writing.

## Conflict of Interest

The authors declare that the research was conducted in the absence of any commercial or financial relationships that could be construed as a potential conflict of interest.

## Publisher’s Note

All claims expressed in this article are solely those of the authors and do not necessarily represent those of their affiliated organizations, or those of the publisher, the editors and the reviewers. Any product that may be evaluated in this article, or claim that may be made by its manufacturer, is not guaranteed or endorsed by the publisher.

## Footnotes

1 E.g., goal 5 of the UN Sustainable Development Goals aims to “Achieve gender equality and empower all women and girls.”

2 The intervention used the Participatory Action Learning for Sustainability or PALS methodology as a strategy to empower women in the ‘Gender and Youth Empowerment in horticulture Markets project’ (GYEM, 2016–2019), a project implemented by SNV Ethiopia and funded by Comic Relief.

3 Geertz described the practice of thick description as a way of providing cultural context and meaning that people place on actions, words, things, etc. Thick descriptions provide enough context so that a person outside the culture can make meaning of the behavior and phenomena described.

4 Throughout the paper, we call the implementing organization, with its human and material resources to implement participatory methods, ‘the facilitator.’

6 Throughout the paper, we will use the PALS terminology for participants – i.e., champions – as it reflects the social changing role the participants are meant to have within this methodology. First-round or original champions are those participants who were selected to attend the training led by the consultant. They had to, as part of the intervention, each train 5 other people in the methodology, the latter being called the second-round champions.

7 VSLA: Village, Savings and Loan Association – these are informal savings groups.

## Appendix 1 | PALS principles and assumptions

PALS is the acronym for Participatory Action Learning for Sustainability. It was developed by the consultant Linda Mayoux as a methodology to empower farmers and increase collaboration among different stakeholders in the value chain. In this methodology, gender inequality is considered a key cause of poverty and women’s empowerment a key strategy for reducing it (Mayoux, 2010). “PALS is not ‘one methodology’ or set of tools. It is a change philosophy based on underlying principles of social and gender justice, inclusion and mutual respect. In particular it promotes women’s human rights based on the United Nations Convention on Elimination of ALL Forms of Discrimination Against Women” (Mayoux, 2018).

In order to do address gender inequality, the PALS methodology catalyzes discussion, reflection and motivation ‘from within’ the participants themselves (Mayoux and De Smet, 2018) rather than imposing ideas from outside. This is to encourage participants to facilitate and monitor the change process themselves so that it would ensure sustainability of the changes toward more gender equality. Through active participation of the poor (inclusion) and encouragement of individual leadership (‘everyone is responsible for its own change’), the participants are guided (facilitation from the back) to express their ideas about how they want to organize their lives and live and work together.

Key principles of the PALS methodology are Mayoux and De Smet (2018):

– **Start with a positive vision:** a strong and positive vision energizes people to make changes toward this vision.
– **Everyone can be a leader:** everyone takes responsibility for their own change.
– **Action from day 1**: every learning ‘event’ focuses on tangible actions for change at individual level, before actions at group and institutional level are designed.
– **Inclusion:** PALS is there to develop the capacities of the poorest and the most vulnerable in order for them to participate equally in development. Often those are the people who need change in order to be able to advance. Them being able to change, is supposed to be inspirational for others to change as well.
– **Facilitation from the back:** facilitation is done in such a way that important issues come from the participants’ own reflection and discussion.
– **Make it fun:** a fun process ensures that “men and women can relax, feel free and happy together which is necessary part of building the movement” and strive for sustainability.

The principles originate from the assumption that if:

– people are led by their own vision and plans for change,
– come together in a fun way and
– can express what they think and feel as individuals,
– with the poorest in the lead,

a change process is initiated and sustained at communal level that will increase gender justice/equality. It becomes apparent from the PALS principles that the methodology is deeply rooted in egalitarian principles and values. This is also reflected in PALS’ stance on gender equality: “women and men treating each other like equal human beings with equal human rights and social responsibilities” (Mayoux, 2018).

This assumption is reflected in the way PALS was organized in rural Ethiopia as part of the GYEM project. The PALS process started with a catalyst phase whereby, in this case, 20 farmers, called ‘champions,’ from five kebeles (villages) participated in a workshop in which they created their individual vision for a happy life. More specifically, women and men identified targets for change and created a road map to reach their vision, starting from their current situation and taking into account opportunities, challenges, strengths and weaknesses. A second session of workshops was held 3–6 months later, being the livelihood strengthening. During this second phase, the achievements were reviewed and more advanced versions of the tools were introduced. The third stage addressed the gender review and the upscaling and sustainability plan. After this process, the champions who completed their training received a certificate. The certificates were handed over during a ‘community open day’ where the peers and neighbors of the champions were invited to be introduced to the tools by the champions and to listen to their experiences. Concurrently, the sustainability and upscaling process started, in which the champions shared their new knowledge with people around them. Next to that, an upscaling training was organized to facilitate the sharing between different woredas (districts). This training was provided by original champions selected based on the proven changes made in their lives and the ability to explain the tools well. At the same time, older men and women champions that did not necessarily comply with these criteria were selected because of their life experience and authority in communities. Since the champions themselves facilitated these workshops, with support of the GYEM staff, a prior refreshment training was also foreseen in which the tools are reviewed (Mayoux and De Smet, 2018).

### Appendix 2 | Recommendations for future empowerment projects using participatory approaches in high power distance countries

#### Mission

Interpret the dynamics in the relationship between facilitator (perceived as high in power), and community (low in power and in need for money and guidance) in the light of power distance. In high power distance cultures, marked by poverty, communities’ missions might be concealed (purposely or not), in order to please the facilitator and maximize the potential win from the intervention. This dynamic in the relationship between facilitator and community will affect the results of the partnership on one hand and will be masked by socially desirable answers on the other.

#### Throughput

Start off with homogenous (power-based) discussion groups to introduce facilitation from the back.

Work with the ‘here and now’ and the dynamics in the room instead of a model. Active participation in the larger groups might not happen as one is used to in more egalitarian settings. One of the tendencies of facilitators is to then ‘correct’ participants, under the apprehension that they do not understand or apply properly the methodology. Instead, facilitators should make room for these differences and abstain from ‘correcting’ participant’s interactions. When space for more discussions is needed in the flow, break the group in small groups: first, homogenous on various dimensions (e.g., gender, age, literacy); then according to random criteria, to mix the groups (e.g., random counting). Using a mix of small groups (at first the same groups, to build confidence and psychological safety, then different groups) and large group setting, in an incremental approach, can help to implement a participative methodology in high power distance settings.

Be realistic in how far these participative methodologies can go in a context where power distance is high and hence active participation goes against what people have internalized. Do not hold the process accountable to standards that were built in a different cultural context and appreciate small progress.

#### Outcome

As facilitators of women empowerment processes, (1) question the evaluation criteria used to assess the success of an intervention, and (2) match the missions of both the facilitator and receiving community in an equal way, instead of assuming that the mission and desired outcomes of the facilitator are more relevant. This requires intensive stakeholder management from the part of the facilitator toward both the community and the donor bodies.

As donor, understand that the definition (Western-based) and operationalization of success criteria (i.e., quantifiable units) are not conducive to an accurate assessment of outcomes.

Since the mission of the community lies more in the economic sphere, as shown in the reported changes, actively seek connection between the PALS activities and interventions in that economic sphere rather than operate as a stand-alone intervention. This symbiosis would increase the long-term impact of the intervention by first addressing the community’s goals and bringing attitudinal change along the way.

#### Impact

Bring in gender as an explicit topic when people start addressing the issue themselves. The Happy Family Tree proved to be a good starting point with concrete changes both men and women wanted to make. Build time and space to go more in-depth in those suggested changes in sex-disaggregated groups first. This can be done through new associative structures that bring women together around an economic incentive (like savings groups) where women come together for an economic incentive (which matches their mission) but are held together because of a social agenda (Kabeer, 2011).

Building on the previous recommendation, create separate structures outside the workshops and the project offering women a reflective space to reassess and redefine their identity within the given power structures and determine their own conceptualization and agenda for gender equality, leading to a social agenda with gender goals defined by women themselves (Kabeer, 2011). Previous research in Bangladesh (Kabeer, 2011) proved that the creation of such reflection spaces that allow conversation around non-traditional identities (so women seeing themselves as agents of their lives, with tools to change their future or their children’s future), strengthens the process of empowerment by allowing the transformation of women’s identity and self-image. Such reflection groups allow women to actively pursue a vision for their daughters’ lives being less influenced by traditional gender expectations and hierarchies.

Apply a longer timespan to assess impact to reveal whether for instance aspirations are followed through with actions by participants (e.g., investing in daughters’ education; delayed marriage age etc.).
